# RGB Imaging as a Tool for Remote Sensing of Characteristics of Terrestrial Plants: A Review

**DOI:** 10.3390/plants13091262

**Published:** 2024-04-30

**Authors:** Anastasiia Kior, Lyubov Yudina, Yuriy Zolin, Vladimir Sukhov, Ekaterina Sukhova

**Affiliations:** Department of Biophysics, N.I. Lobachevsky State University of Nizhny Novgorod, 603950 Nizhny Novgorod, Russia; nastay2903@bk.ru (A.K.); lyubovsurova@mail.ru (L.Y.); uchebnayap.zolin@gmail.com (Y.Z.); vssuh@mail.ru (V.S.)

**Keywords:** RGB imaging, color models, color indices, multispectral imaging, hyperspectral imaging, plant characteristics, plants, remote sensing, water deficit

## Abstract

Approaches for remote sensing can be used to estimate the influence of changes in environmental conditions on terrestrial plants, providing timely protection of their growth, development, and productivity. Different optical methods, including the informative multispectral and hyperspectral imaging of reflected light, can be used for plant remote sensing; however, multispectral and hyperspectral cameras are technically complex and have a high cost. RGB imaging based on the analysis of color images of plants is definitely simpler and more accessible, but using this tool for remote sensing plant characteristics under changeable environmental conditions requires the development of methods to increase its informativity. Our review focused on using RGB imaging for remote sensing the characteristics of terrestrial plants. In this review, we considered different color models, methods of exclusion of background in color images of plant canopies, and various color indices and their relations to characteristics of plants, using regression models, texture analysis, and machine learning for the estimation of these characteristics based on color images, and some approaches to provide transformation of simple color images to hyperspectral and multispectral images. As a whole, our review shows that RGB imaging can be an effective tool for estimating plant characteristics; however, further development of methods to analyze color images of plants is necessary.

## 1. Introduction

Plants play an important role for life on Earth, providing global productivity and participating in water exchange and climate formation [1]; particularly, they are sources of raw materials and food for humanity. Monitoring plants is important for their protection under changeable environmental conditions and increasing productivity [2]. Particularly, the monitoring of agricultural plants provides tracking growth and development rates, prediction of biomass and crops [3,4,5,6], management of application of fertilizers [7,8,9] and phytohormones [10], detection of biotic [11,12] and abiotic [13,14] stressor actions, and others. In natural ecosystems, plant monitoring can be additionally used to observe the compositions of species and the dynamics of their areas that are important for the protection of these ecosystems [15,16]. Plant monitoring in cities can be important for creating comfortable environmental conditions, managing pollution, and others.

Plant monitoring based on optical methods is distant and relatively simple; these methods can be used for large areas and, therefore, provide fast remote sensing of plant characteristics [17]. It is known that interaction with plants can qualitatively change light spectra; this effect is related to the absorption of visible light by plant pigments (mainly photosynthetic pigments), the absorption of short-wave infrared light (SWIR) by water, and the scattering of near-infrared light (NIR) by the internal structures of leaves [17,18]. Additionally, the fluorescence in the red and far-red spectral ranges can inform on photosynthetic activity and its changes under the actions of stressors [19,20,21].

It is important that physiological processes in plants can be related to narrow spectral bands, because different pigments or their different transitive forms absorb light and, in some cases, emit fluorescence in individual spectral bands [22,23,24,25]. Different chlorophylls [20,21], epoxidated and de-epoxidated forms of xanthophylls [26], or phytochrome forms [24] are important examples of pigments influencing the spectra of light absorption in plants. As a results, the value of reflectance [27,28], positions of its extremums (e.g., water reflectance minimum [27] or pigment reflectance maximum [17,28]), and reflectance slope (e.g., red edge [29]) are sensitive to physiological changes in plant. As a result, different plant characteristics, including their changes under the actions of stressors, have specific spectral signatures of reflectance and can be used for the remote sensing of plants.

Hyperspectral and multispectral imaging provide information about the reflectance of plants through measuring reflectance in a series of narrow spectral bands (forming reflectance spectrum) and in several specific bands, respectively; it is measured in each pixel of the image [30]. Using narrowband reflectance indices, which are based on hyperspectral and multispectral imaging of plants, is a perspective tool for the estimation of plant characteristics and their changes under the actions of stressors. Particularly, changes in the reflectance indices can be related to changes in the content of photosynthetic pigments [23,29], water [27], nitrogen [17], leaf area index (LAI), biomass [17,28], primary productivity [31], and other plant characteristics. Despite the advantages of hyperspectral and multispectral imaging for plant remote sensing, using this imaging has some serious restrictions. Hyperspectral imaging is technically complex and needs high-cost measuring systems (hyperspectral cameras); using a hyperspectral camera in remote sensing requires strong synchronization between measurements and movement of the used mobile platform (e.g., unmanned aerial vehicle, UAV) [32]. There are technologies (e.g., snapshot technology) that can additionally increase the velocity of hyperspectral measurements; however, the technical complexity and cost of these cameras are also strongly increasing [32,33]. Multispectral imaging is simpler and more accessible for plant remote sensing; however, multispectral cameras remain relatively expensive [28].

In contrast, RGB imaging, which is based on the primary measurement of reflectance in red (R), green (G), and blue (B) spectral bands, is technically simple and widely accessible for plant remote sensing. Digital cameras with the matrix equipped by the Bayer filter (RGB cameras) are used for this imaging [34]. The matrix of RGB cameras includes 25%, 50%, and 25% pixels with red, green, and blue light filters, respectively [34]. As a result, values of R, G, and B are directly measured or are calculated by interpolation based on corresponding values in the nearest pixels [34]. These systems do not require the application of a prism (or diffraction grating) and “line by line” scanning, which are widely used in hyperspectral cameras, or the application of several elementary cameras equipped with narrowband spectral filters, which are widely used in multispectral cameras [28]. However, using interpolation for the color value calculation can increase spatial resolution requirements in comparison with monochromic cameras. The simplicity of the RGB camera contributes to using various platforms for RGB imaging [35,36,37,38,39], including handheld devices (e.g., photo cameras or mobile phones) used for small and moderate distances from plants or plant canopies, land transport (e.g., tractors) used for moderate distances, UAV (copter and drones) used for moderate and large distances, airplanes used for large distances, and satellites used for extremely large distances. Increasing the distance from plants accelerates imaging [28] but decreases the spatial resolution and makes it difficult to exclude backgrounds.

However, using RGB imaging has important restrictions. Particularly, the plant reflectance in red, green, and blue spectral bands has relatively small differences, e.g., the difference between the reflectance for NIR and red light is higher than the difference between the reflectance for red and green light [40,41]. As a result, plant remote sensing based on measuring the reflectance in both NIR and visible light spectral bands is often better at showing plant characteristics than the sensing based on visible spectral bands only [38,42,43,44]. However, there are works showing similar efficiencies of both methods [36,45]. Particularly, some works show that the analysis of RGB spectral bands can have a high efficiency for the estimation of plant biomass and chlorophyll content [41,44].

In spite of the fact that color changes can indicate stages of plant development, maturation, senescence [11,13,46], shortage of fertilizations [7,9], action of stressors [13,14], etc., simple observations of color images are weakly informative and can only show strong changes in plants. Thus, the development of methods of analysis of color images is an important way to increase the efficiency of plant RGB imaging. Calculations of non-dimensional color indices [44,47], texture analysis [48,49], and machine learning [14,50,51] are widely used to estimate plant characteristics (including the contents of chlorophylls and carotenoids, biomass and LAI, nitrogen plant content, and others) based on color images. These plant characteristics are widely investigated because they can be used to estimate plant development and growth rate under different environmental conditions. Analysis of color images can also be used for the estimation of characteristics of the crops of agricultural plants, e.g., quantity of flowers [52], grain yield [3,4], or seed quality (protein and oil content [53]). Some works additionally show the perspectives of analysis of color images for estimations of water content, canopy temperature, canopy-to-air temperature difference, sap flow, and other characteristics [54,55].

Another important direction of investigations in plant RGB imaging is the development of methods of transformation of color images to multispectral and hyperspectral images [56]. At present, these methods are mainly preliminary; however, they could strongly simplify plant remote sensing in the future.

Considering the potential importance of using plant RGB imaging, the aim of our review was to summarize the literature data about the practical application of this RGB imaging for the remote sensing of plant characteristics. This review focused on the analysis of information on widely used color models, methods of background exclusion, ways of using RGB imaging for the estimation of plant characteristics, and approaches for the transformation of color images to multispectral and hyperspectral images. We did not review numerous works (see, e.g., reviews [11,12]) devoted to the actions of phytopathogens on plant color parameters because it is a separate and very extensive problem.

## 2. Widely Used Color Models

The analysis of color images, which are provided by RGB imaging, requires consideration of the colorimetry basis and widely used color models. Hue, saturation, and brightness are the basic parameters that provide information on color in the colorimetry. The hue shows the type of chromatic color in the visible light spectrum, including violet, blue, cyan, green, etc.; white, grey, and black, which are achromatic colors, are not included. The degree of chromaticity is shown by the saturation of color. The achromatic color has 0% saturation, and the fully chromatic color has 100% saturation. Brightness is relative lightness that ranges from 0% (black) to 100% (white) [57].

**Figure 1:**
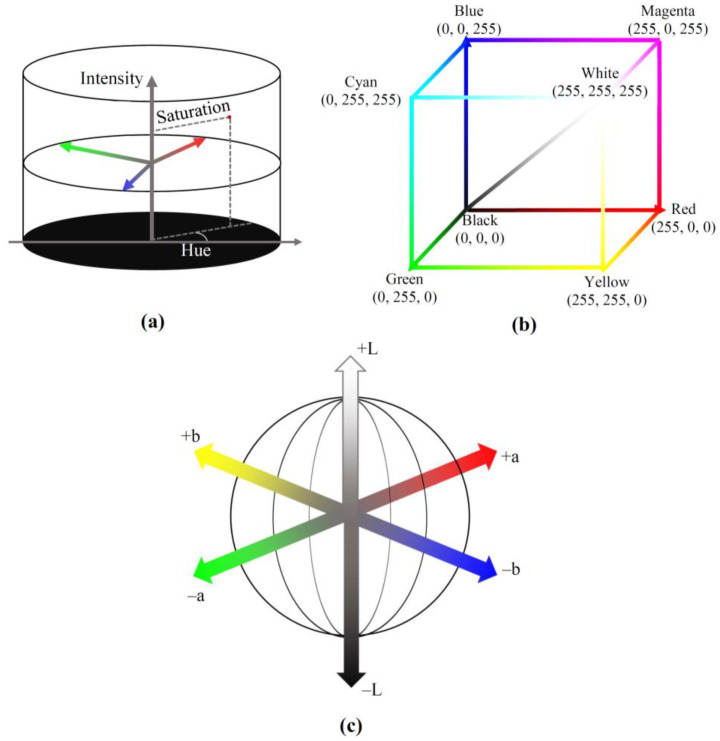
Schemes of HSI (hue, saturation, intensity) (**a**); RGB (red, green, blue) (**b**); and L*a*b* (brightness, red-green, yellow-blue) (**c**) color models. Typical range of R, G, and B (from 0 to 255) is shown. The L*a*b* and L*u*v* color models are initially proposed for the industrial application of colors. These spherical models include the normalized brightness axis (L*) and red–green (a* or u*) and yellow–blue (b* or v*) chromatic axes (Figure 1c) [59]; all coordinates are calculated based on X, Y, and Z (see above) and X^0^, Y^0^, and Z^0^, which are values of X, Y, and Z using the white reflectance calibration standard (Table 1). The combination of these chromatic coordinates determines all colors [61,62]. The system L*c*h* is based on other color components, including the brightness, chroma, and hue [61], which are calculated based on L*, a*, and b* in the L*a*b* model (Table 1).

There are several color models that are actively used for the description of color images (Table 1, Figure 1). HSB (hue, saturation, brightness) and similar HSI (hue, saturation, intensity) models strongly correspond to the colorimetry basis [57] and can be effectively used in RGB imaging (Figure 1a). Particularly, the hue is weakly affected by light conditions and shadows [58]; as a result, using the HSI model provides effective segmentation and contrasting of objects in color images [11,58].

However, registration of color images is based on using RGB cameras with matrices equipped by the Bayer filter (red, green, and blue filters), meaning that the HSB and HSI models do not technically correspond to this registration. In contrast, the RGB (red, green, blue) model strongly corresponds to using RGB cameras because R, G, and B are absolute chromatic coordinates showing intensities of light in red, green, and blue spectral channels, respectively (Figure 1b). The combination of R, G, and B determines hue, saturation, and brightness of color [49,59], providing, e.g., calculation of coordinates of HSB and HSI color models (Table 1). The normalization of RGB (the normalized rgb color model) decreases the influence of brightness, surface orientation, and other factors on the parameters of color images [60].

**Table 1:** Color models widely used in investigations.

Color Model	Color Model Transformation	Description
RGB [49,61]		R, G, and B are absolute chromatic coordinates for red, green, and blue colors, respectively; they determine hue, saturation, and brightness.
		Values of R, G, and B are initially measured by matrix of RGB camera and typically range from 0 to 255.
rgb [58,59]	$r=\frac{R}{R+G+B}$ $g=\frac{G}{R+G+B}$ $b=\frac{B}{R+G+B}$	r, g, and b are normalized chromatic coordinates for red, green, and blue colors, respectively.


HSI (variant 1) [58]	$Hue=\frac{\sqrt{3}\left(G-B\right)}{\left(R-G\right)+\left(R-B\right)}$	Hue, saturation, and intensity are standard color characteristics.
	$Saturation=1-\frac{3}{R+G+B}(\min\left(R,G,B\right))$	
	$Intensity=\frac{R+G+B}{3}$	
HSI (variant 2) [60]	$Hue=\left\{\begin{array}{c} \theta \;\;\;\;\;\;\;\;\;\;\mathrm{if}\;B\leq G\\ 360-\theta \;\mathrm{otherwise} \end{array}\right.$ $\theta ={cos}^{-1}\left\{\frac{\frac{1}{2}[\left(R-G\right)+(R-B)]}{[{\left(R-G\right)}^{2}+\left(R-B\right)(G-B){]}^{\frac{1}{2}}}\right\}$	Hue, saturation, and intensity are standard color characteristics.
	$Saturation=1-\frac{3}{R+G+B}(\min\left(R,G,B\right))$	
	$Intensity=\frac{R+G+B}{3}$	
HSB [37,60]	$Hue=\left\{\begin{array}{c} 60\cdot \left\{\frac{G-B}{\max\left(R,G,B\right)-\min\left(R,G,B\right)}\right\},\;\mathrm{if}\;\max\left(R,G,B\right)=R\\ 60\cdot \left\{\frac{B-R}{\max\left(R,G,B\right)-\min\left(R,G,B\right)}\right\},\;\mathrm{if}\;\max\left(R,G,B\right)=G\\ 60\cdot \left\{\frac{R-G}{\max\left(R,G,B\right)-\min\left(R,G,B\right)}\right\},\;\mathrm{if}\;\max\left(R,G,B\right)=B \end{array}\right.$	Hue, saturation, and brightness are standard color characteristics.
	$Saturation=\frac{\max\left(R,G,B\right)-min(R,G,B)}{\max\left(R,G,B\right)}$	
	Brightness=max⁡(R,G,B)	
XYZ [57,58,61,62]	*Transformation from RGB to CIE XYZ* X=0.607·R+0.174·G+0.200·B Y=0.299·R+0.587·G+0.114·B Z=0.066·G+1.116·B	Y is the brightness. X and Z are virtual components of the primary spectra, where Z is related to short-wavelength light and X is related to large- and medium-wavelength light.
	*Transformation from rgb to CIE XYZ* X=0.412·r+0.358·g+0.180·b Y=0.213·r+0.751·g+0.072·b Z=0.019·r+0.119·g+0.950·b	
	X/X^0^ > 0.01, Y/Y^0^ > 0.01, Z/Z^0^ > 0.01	X^0^, Y^0^, and Z^0^ are values of X, Y, and Z using the white reflectance standard
L*a*b* [49,58,61]	$L^{*}={116\left(\frac{Y}{Y^{0}}\right)}^{\frac{1}{3}}-16$	L* is the normalized brightness.
	$a^{*}=500\left({\left(\frac{X}{X^{0}}\right)}^{\frac{1}{3}}-{\left(\frac{Y}{Y^{0}}\right)}^{\frac{1}{3}}\right)$	+a* is chroma in redness. −a* is chroma in greenness.
	$b^{*}=200\left({\left(\frac{Y}{Y^{0}}\right)}^{\frac{1}{3}}-{\left(\frac{Z}{Z^{0}}\right)}^{\frac{1}{3}}\right)$	+b* is chroma in yellowness. −b* is chroma in blueness.
L*u*v* [49,58,61]	$L^{*}={116\left(\frac{Y}{Y^{0}}\right)}^{\frac{1}{3}}-16$	L* is the normalized brightness.
	$u^{*}=13L^{*}\left(\frac{4X}{X+15Y+3Z}-\frac{4X^{0}}{X^{0}+15Y^{0}+3Z^{0}}\right)$	+u* is chroma in redness. −u* is chroma in greenness.
	$v^{*}=13L^{*}\left(\frac{9Y}{X+15Y+3Z}-\frac{9Y^{0}}{X^{0}+15Y^{0}+3Z^{0}}\right)$	+v* is chroma in yellowness. −v* is chroma in blueness.
L*c*h* [49,61]	$L^{*}={116\left(\frac{Y}{Y^{0}}\right)}^{\frac{1}{3}}-16$	L* is the normalized brightness.
	$c^{*}=\sqrt{{\left(a^{*}\right)}^{2}+{\left(b^{*}\right)}^{2}}$	c* is the chroma.
	$h^{*}=arctan\left(\frac{b^{*}}{a^{*}}\right)$	h* is the hue.

The XYZ color model imitates the perception of light by the human retina, namely S-cones (reception of short wavelength), M-cones (medium wavelength), and L-cones (large wavelength) [58,59]. Y corresponds to brightness, and X and Z are virtual values of the primary spectra [61]. Z is related to short-wavelength light, and X is related to large- and medium-wavelength light [59]. X, Y, and Z are calculated based on R, G, and B in accordance with the equations shown in Table 1.

Thus, there are various color models (some of which are considered above) focusing on different characteristics of colors (e.g., hue, saturation, and brightness of plant color can be independently investigated based on the HSB color model). It can be expected that using various color models provides different efficiencies of detection of specific changes in plants, i.e., using the optimal color model is one of the methods of analysis of color images in plant remote sensing.

## 3. Methods of Background Exclusion

The development of methods of separation between plant canopy and background (mainly soil background) is an important task for plant remote sensing because necessity of this separation is supported by experimental works; particularly, Scharf and Lory [63] revealed that excluding soil pixels increases the relation of color parameters to nitrogen (N) content and SPAD (which shows the content of chlorophylls). However, the great variability of color and texture characteristics of the background (e.g., various soils) can disturb the separation between the plant canopy and the background [64,65].

The analysis of images, which include plants and background objects (water, bare lands, roads, buildings, etc.), shows that they have significant differences in red and green spectral bands [39]. In contrast, differences in the blue band can be weak (e.g., for plants and water). Ref. [39] showed that the analysis of reflectance in red and green spectral bands can be effectively used for the separation of plants from other objects. In contrast, Woebbecke et al. [64] showed that soil reflectance in the red band is higher or similar to the reflectance of plants, and the reflectance in the blue band is high for most soil surfaces. The reflectance in the green band is related to plants [39,64]. These results show that R, G, and B can be the basis of separation between plants and backgrounds; however, the efficiency of simply using reflectance in these bands for separation is rather restricted.

Color indices, which are based on the combination of reflectance in R, G, and/or B, are widely used for the separation between plants and background (particularly soil) [64,65,66,67], and separation is often based on using the threshold methods. Particularly, it was shown that using the excess green index (ExG) forms near-binary images which provide the separation between plants and background [64]. Meyer and Neto [65] showed that the difference (ExGR) between the excess green index (ExG) and excess red index (ExR) can be effectively used for the separation between soybean plants and two types of background (soil and straw). The difference between G and R (GMR) is also used for separation between plants and soil background [68,69].

The threshold method of the separation between plants and background in color images is widely used. It is based on the assumed threshold [70,71,72] or on the estimated threshold, which can be revealed through using the Otsu-based method [65,73], Ridler method [74,75], triangle method [76], histogram-based methods [77,78,79,80], and other methods of object classification.

There are numerous examples of using color indices for the separation between plants and background. Kataoka et al. [73] used the color index of vegetation (CIVE) for separation between plants and background; the threshold level was calculated based on discriminant analysis. Kırcı et al. [81] estimated the threshold based on color indices and histograms of their distributions; scatter plots were used to classify vegetation and soil. Netto et al. [76] compared methods of estimation of thresholds for normalized difference index (NDI), ExGR, and ExG. It was shown that the triangle, Otsu-based, and Ridler methods were very effective and had high accuracy (about 85–90%). Zhang et al. [39] used the iterative method to estimate the optimal threshold for the separation between background and vegetation pixels.

The new histogram-based method of the threshold estimation was suggested by Liu et al. [80]. In accordance with this method, the frequency histogram of index values (ExG or a* from the L*a*b* color model), which are related to plants and background, are fitted by the sum of Gaussians. Three variants of thresholds are further calculated. The initial threshold (T_0_), which approximately shows borders between soil and plant pixels, is estimated based on the visual minimum between maximums, related to plants and background, in the frequency histogram of ExG (or a*). T_1_ and T_2_ thresholds are based on the calculation of ExG (or a*) corresponding to the intersection of Gaussians related to plants and background. Calculation of the T_1_ threshold is based on the minimization of errors; calculation of the T_2_ threshold is based on the assumption of equal errors for plants and background. It is interesting that the T_2_ threshold seems to be the most effective in the separation between plants and background. Song et al. [82] proposed to use this histogram-based method in combination with the HSI (hue, saturation, intensity) color model for revealing shaded parts of plants. Zhang et al. [55] proposed to use the mean value of Gaussian distribution of ExG for a maize canopy (MGDEXG) to exclude the background.

It should be finally noted that excluding parts of plants from the image can increase relations between measured color indices and plant characteristics. Particularly, Liu et al. [83] showed that excluding naked barley ears in the color image can improve relations between some color indices and SPAD.

Thus, different reflectances of plants and background can be used to exclude this background. Exclusion is the basis of further analysis of the color images of plants to estimate their characteristics.

## 4. Estimation of Plant Characteristics Using RGB Imaging

### 4.1. Content of Photosynthetic Pigments and Nitrogen Content

We consider the relation of color parameters to the content of photosynthetic pigments (chlorophylls a and b, carotenoids) and to the nitrogen content in the same Section 4.1 because the content of chlorophylls in plants is strongly related to the nitrogen content [84]. It should be noted that optical SPAD chlorophyll meters, which are based on the measurements of light transmission through leaves at 650 and 940 nm [85], are widely used for the estimation of the content of chlorophylls a and b, concentration of carotenoids, and nitrogen content [86,87,88]. Thus, the analysis of relations between color parameters and SPAD values is also included in this section.

Chlorophylls are known to strongly absorb light in red and blue spectral bands of the visible light [20,89]; in contrast, green light is minimally absorbed by chlorophylls and can be used by photosynthetic processes in deep layers of the leaf [90]. It can be expected that the chlorophyll content should be related to the reflectance in these spectral bands; however, these relations can be intricated.

It is known that reflectance in the red and green spectral bands is negatively correlated to concentrations of chlorophylls a and b and total chlorophylls; in contrast, reflectance in the blue spectral band has a positive linear correlation with the chlorophyll concentrations [37,53,91,92,93,94]. The relation between B and the chlorophyll content can be non-stable [95]; this relation is stronger for the normalized b values [93,96]. The investigation by Gupta et al. [97] showed that the relation between SPAD values and non-normalized R, G, and B were low and not significant; in contrast, using the normalized r, g, and b was more efficient. The chlorophyll content is also related to parameters of other color models, including HSI, HSB, and L*a*b* [37,91,93] (Table 2); however, the strength of these relations can be moderate in some cases (e.g., R^2^ is about 0.38–0.39 for relations between the content of photosynthetic pigments and a* and 0.48 for the relation between the total chlorophyll content and the hue [37]).

The relations of R, G, and B to the nitrogen concentration are similar to their relations to chlorophyll contents, i.e., high and negative correlations are observed for R and G, and a low correlation is observed for B [91]. However, Mercado-Luna et al. [100] showed that R and B can be negatively correlated with the nitrogen content in plants; in contrast, G is weakly related to this content. The sign of correlation coefficients between the nitrogen content and color coordinates (R, G, B, r, g, and b) can be dependent on the stage of development [96,104]; these dependences, potentially, explain the confused results described above.

The analysis of other color models shows similar results, e.g., the relations of b* (L*a*b* color model) to the leaf nitrogen concentration and SPAD differ at different stages of plant development [134]. Leaf thickness and plant cultivar can also influence these relations [134].

However, the simple analysis of coordinates in different color models has limited efficiency for the estimation of the concentration of photosynthetic pigments and nitrogen content. There are complex analyses of color parameters that provide increased efficiency of this estimation.

It is known that ExG, VARI (visible atmospherically resistance index), GLI (green leaf index), simple and normalized ratio indices, DGCI (dark green color index), and others can be strongly related to SPAD, chlorophyll concentration, or nitrogen content (Table 2). Particularly, color indices can be used for the estimation of nitrate concentrations in leaves [105] and whole plants [104,105] and for the estimation of these concentrations in soil [106]. It should be noted that using color indices, which are simultaneously based on R, G, and B, can be more effective for the estimation of nitrogen and chlorophyll content than using the indices, which are based on two spectral bands [126]. The last point is supported by the efficiency of the application of RGFI (red–green fit index) and BGFI (blue–green fit index) for SPAD estimation in potato [119]; both indices are calculated by using all R, G, and B and fitting-based constants.

Using regression models, which describe dependences of concentrations of chlorophylls and SPAD on color parameters, is another way to analyze images. Riccardi et al. [98] showed that single (based on R or G) and multiple (based on R, G, and B) regression models can be effective tools for estimating chlorophyll content by using plant RGB imaging and have low noise. Ge et al. [104] showed that regression models, which are based on using both color indices and color moments, can be the most effective for estimating plant nitrogen concentration.

PCA (principal component analysis) can be additionally used to increase the efficiency of analysis of color images. It is known [101] that PCA-based components can be the basis of regression models estimating nitrogen content in plants. On the other hand, PCA is a powerful instrument for the construction of new color indices, e.g., I_PCA_, which can be used to estimate SPAD [114,126].

There are other approaches that can be used to estimate the concentrations of chlorophylls, nitrogen content, and SPAD. Particularly, Wiwart et al. [137] showed the high efficiency of using Euclidean distances between parameters of HSI or L*a*b* color models for the detection of N and Mg deficiencies in plants.

Methods of texture analysis are the next group of methods to estimate plant characteristics based on RGB imaging. Particularly, Chen et al. [99] showed that the texture parameters of leaves (including mean, median, and skewness parameters) can be effectively used for the estimation of SPAD through the development and application of regression models describing dependences of SPAD on these parameters. Additionally, Fu et al. [49] showed that Gaussian process regression using Gabor-based textures provides high accuracy for the estimation of plant nitrogen density; in contrast, partial least square regression using gray level cooccurrence matrix-based textures is optimal for the measurement of plant nitrogen concentration.

Color indices can also be used as input variables for machine learning to provide effective estimations of SPAD [112], chlorophyll content [51], and plant and leaf nitrogen concentrations [105,138]; the efficiency of machine learning (at least, for the estimation of nitrogen in plants) can be decreased by increasing the plant growth stage [105]. The combination of texture parameters and color indices can be also used as input variables of neural networks with back propagation; using this combination increases the efficiency of chlorophyll concentration estimation in comparison with using only color indices for the machine learning [51].

Finally, it should be noted that changes in the concentration of carotenoids can also be related to the parameters of color images. It is known that carotenoids mainly absorb blue and green light [23]; in contrast, chlorophylls mainly absorb blue and red light. The actions of stressors or senescence induce degradation of both chlorophylls and carotenoids; however, carotenoid degradation is slower [22]. Thereby, the changes in reflectance in red and blue spectral bands should be different [22,28], i.e., changes in the parameters of color images should also be sensitive to the carotenoid concentration. It was shown [37] that G is negatively related to the concentration of carotenoids (R^2^ is 0.67); in contrast, R was moderately related to this concentration (R^2^ is 0.45). Using HSB and L*a*b* color models, the calculation of DGCI additionally showed the relation of the parameters of color images to the content of chlorophylls and carotenoids [37]. Widjaja Putra and Soni also showed that the carotenoid concentration is correlated with some additional color indices [44].

As a whole, RGB imaging can be used to estimate concentrations of chlorophylls and carotenoids in plants, their nitrogen content, and SPAD (which is widely used as the simple characteristic of the chlorophyll content in plants).

### 4.2. Plant Development and Productivity

Remote sensing of plant productivity can be based on the estimation of its biomass, which is related to the growth rate, nutrition status, grain yield, and other characteristics [116,129], or its LAI, which is related to light absorption [139] and the biomass production rate [140]. LAI is also used as the important variable in models for estimation of the CO_2_ assimilation and water exchange [141,142].

It is known that R can be strongly related to the fresh and dry biomass and LAI [101]; G is also related to the biomass [66]. This result is in good accordance with the relations of R and G to the content of chlorophylls (Table 2) because light absorption by chlorophylls plays a key role in the photosynthesis and, thereby, productivity of plants.

It is known that numerous color indices (including GLI, GR (green/red simple ratio), NGRDI (normalized green/red difference index), VEG (vegetative index), and others) can be strongly related to the plant biomass and can be used for its estimation [6,50,116,122,129]. Leaf overlapping can disrupt relations between color indices and the plant biomass [69], e.g., this effect is observed using NGRDI [40]. There are numerous color indices including GMR, simple ratios, NDRBI (normalized difference red/blue index), NExG (normalized excess green index), ExR, NGRDI, VARI, and VEG [69,101,117,124] that are strongly related to LAI and can be used for its estimation; however, leaf overlapping can also disrupt these relations [69]. Finally, it is interesting that the plant height and stem diameter [101,125] can also be estimated based on color indices (see Table 2 for detail).

There are several ways to increase the efficiency of using color indices to estimate plant biomass. Particularly, using the canopy volume model, which is based on the simultaneous measurements of color indices and the structural characteristics of the canopy (e.g., height and pixel area), provides an effective biomass estimation [5]. It is important to note that using color indices to estimate aboveground plant biomass is more effective than using narrowband reflectance indices [50].

Using a multiple stepwise regression technique based on the measurements of color indices and texture parameters also increases the accuracy of plant biomass estimation [6]; investigations using only color indices or only texture parameters are less effective. Color indices can be used as input variables for machine learning to estimate plant biomass [6,50]; analysis of the combination of color indices and narrowband reflectance indices is more effective for plant biomass estimation (e.g., using the random forest model [50]). Finally, it should be noted that the PCA is a powerful instrument for the construction of new color indices that can be used to estimate plant biomass, LAI, and height [5,101].

Plant yield is another characteristic that is strongly related to plant productivity. It is known that plant yield can be strongly related to color indices, including, e.g., VARI and DGCI (Table 2). The relations between color indices and grain yield can be dependent on the development stage, e.g., the booting stage [124], filling stage [4], or stage after flowering [143] are optimal for the prediction of the yield based on color indices.

The sensitivity of color indices to the grain yield is mainly based on the dependence of this yield on the nitrogen content [3,4,38,107,109], which plays a key role in the grain formation and is strongly related to color parameters (see Section 4.1 and Table 2). Additionally, the nitrogen content influences the grain quality and nutritional value [144,145], i.e., these characteristics can also be related to color parameters. Vollmann et al. [53] showed that R and G are moderately and negatively related to protein and oil content in soybean seeds (R^2^ is about 0.45–0.65); in contrast, B is positively correlated with these characteristics (R^2^ is about 0.45–0.62) (see Table 2 for details).

These results are the basis for the development of nutrition management based on RGB imaging. Yuzhu et al. [106] showed that g is negatively related to the total content of nitrogen in plants, nitrate concentration of leafstalk, content of inorganic nitrogen in soil, and SPAD (R^2^ is about 0.58–0.76); as a result, remote sensing of this normalized parameter can support the timely use of nitrogen fertilizers to contribute to the maximal yield of the plant (pepper). There are other examples of nutrition management increasing the plant yield. Leaf color and textures can be used in the fuzzy K-nearest neighbor classifier [8] to estimate deficits in mineral nutrient elements; parameter a* from the L*a*b* color model can be used to timely reveal N, P, Mg, and Fe deficits [7].

Estimation (or prediction) of the plant yield can be based on the more complex analysis of color images. Particularly, plant quantity, plant height, and color parameters (G, B, R/B, (G-B)/(R-G), VARI, and GLI) can be used as input values for multiple and stepwise regression models to estimate plant yield [146]. The application of color indices and texture parameters as input for the RFE_ELM model can be effectively used to estimate cotton yield [147]. The complex analysis of color indices by using the crop surface model and linear regression model provides a prediction of corn yield [75]. Using color indices at two different stages of plant development (at the booting and jointing stages for VARI) in the multiple linear regression model increases the efficiency of grain yield prediction [124]. Using a combination (sum) of color indices (NGBDI, GR, and ExG) in the regression model can also be very effective for estimating grain yield [143]. Finally, it should be noted that the remote sensing of flower formation can be additionally used for the prediction of plant yield. Wan et al. [52] showed that color indices correlate with flower number and can be used as the input in the random forest or optimal subset regression model to provide the yield prediction.

Remote sensing of plant development can be based on the biomass and yield estimation; however, there are other estimators of this process. Particularly, the development induces color changes in the leaves of plants [13,46], i.e., the plant greenness increases from spring to summer, and the red reflectance band prevails in the autumn (as a result of leaf senescence). This dynamic is related to changes in the ratio of concentrations of chlorophylls, carotenoids, and anthocyanins caused by seasonal plant development [22,46]. This effect is lower in evergreen plants (particularly, coniferous trees) [46]; however, the dynamics of ExG have the summer maximum for both types of plants. It is interesting that the seasonal dynamics of ExG are strongly correlated with the global primary productivity of plants [46].

Finally, RGB imaging can also be used for revealing leaf senescence caused by the actions of environmental stressors. It is known that the actions of many abiotic stressors induce leaf redness [13]. Particularly, Adamsen et al. [113] investigated the senescence rate of wheat under elevated CO_2_ and limited soil nitrogen. It was shown that GR can be used for estimation of the senescence rate because this color index is related to the quantity of leaves, which decreases under senescence, and SPAD, which is dependent on the concentration of chlorophylls and, thereby, is also sensitive to leaf senescence.

As a whole, RGB imaging can be effectively used for remote sensing of plant productivity, yield, and development. Particularly, this imaging provides revealing plant stress changes caused by deficits of nutrients (especially, nitrogen) and supports the timely use of fertilizers.

### 4.3. Plant Changes Induced by Water Deficit

Water deficit, which is caused by drought and salinization, is the key abiotic stressor influencing terrestrial plants. A water deficit decreases the productivity of plants and can induce their death. Stomata closure accompanying this deficit suppresses photosynthesis and increases leaf temperature [148]. It means that the remote sensing (including RGB imaging) of plant changes due to water deficits is an important applied problem.

It is known that the water stress level can be detected based on the color coordinates in the HSI, RGB, and rgb color models (Table 2) [13] because the water deficit causes plant senescence, which leads to a significant prevalence of reflectance in the red spectral band over reflectance in the green and blue bands [22,46]. A great fraction of red reflected light is not typical for leaves of plants under favorable environmental conditions [13]. However, these simple methods of water deficit detection have limitations because these changes in leaf color can be induced by other reasons (e.g., leaf senescence is observed during seasonal changes in plants [13]). Thus, further development of methods of water deficit detection based on plant RGB imaging is important.

The color index MGDEXG (the mean value of the Gaussian distribution of the excess green index) can be used for revealing water deficits in plants; a decrease in this index shows insufficient irrigation [55]. It is known that MGDEXG is strongly related to the leaf water potential and sap flow during the late vegetation stage, reproductive stage, and maturation stage of plant development [55].

It is interesting that MGDEXG (and NExG, Table 2) is strongly related to the crop water stress index (which is used in thermography as an indicator of water stress [149]) and to canopy temperature [55]. The canopy temperature is also related to RGRI (red/green ratio index) [54]. These results show that the canopy temperature and, probably, the crop water stress index can be estimated based on color images, i.e., plant RGB imaging can be potentially used for revealing stomata closure caused by the water deficit.

The texture analysis of color images can also be used for the estimation of plant water status. It is known that the mean and kurtosis of the grayscale values of RGB are strongly correlated with the water content of leaves [48]. It can be hypothesized that changes in kurtosis are possibly related to the increasing heterogeneity of leaf coloring and formation of defects and roughness under water deficit action. Increased color heterogeneity can be caused by heterogeneous chlorophyll destruction; the formation of defects and roughness can be potentially induced by decreasing the water content in the epidermal cells of plant leaves. This is also a potential reason for changes in the mean grayscale values of RGB, because the roughness can influence light scattering from the leaf surface [150].

Machine learning can also be used to detect the action of water deficit on plants based on RGB imaging. Particularly, Zakaluk and Ranjan [14] analyzed color parameters and indices in plants with PCA; the PCA components were used as the input in the artificial neural network-based model. The analysis showed [14] that using principal components in combination with machine learning provided the detection of changes in the leaf water potential and distinguished these changes from changes induced by soil nitrate content.

Finally, there are preliminary arguments supporting the possibility of revealing small and fast changes in water content based on RGB imaging. Our previous works [151,152] showed that changes in the intensity of reflected light in broad spectral bands (about 100 nm, similar to the spectral bands of RGB imaging) and reflectance indices based on these intensities were related to small and fast changes in the water content in leaves under generation and propagation of long-distance electrical signals.

As a whole, RGB imaging seems to be the perspective tool for the detection of water deficit action on plants and the estimation of characteristics of this action.

### 4.4. Variability of Efficiency of Color Parameter Used for Estimation of Plant Characteristics

It should be additionally noted that we did not exclude relations with low determination coefficients from Table 2 in cases ranging R^2^ (R^2^ < 0.36 is shown as a minimum determination coefficient in the table). These relations provide a more accurate analysis of efficiency of using specific parameters of color images for the estimation of specific plant characteristics and exclude misrepresentations of the estimation of their efficiency. Results of the current review show that this efficiency can be strongly varied because determination coefficients for regressions describing relations between the color parameters and plant characteristics are widely ranged (Table 2).

This variability of relations can be observed for regressions that are shown in different investigations and for regressions that are shown in the same investigation (see, e.g., [3,4,9,41,44,69,83,95,101,102,112,114,124,129,132]). Particularly, the determination coefficient for the regression describing the relation of the aboveground biomass to r, g, and GR are 0.05–0.84 [6,96,102], 0.02–0.79 [5,69,96], and 0.01–0.85 [50,69,96], respectively.

These results mean that there are conditions providing effective estimations of plant characteristics based on color parameters; in contrast, estimation can be impossible under non-optimal conditions. Revealing these optimal conditions is an important task of plant remote sensing based on RGB imaging. Particularly, it is known that the efficiency of plant characteristic estimation can be strongly dependent on the growth stage [4,102,104,120,134], and the direction of effect can differ for different color indices. The height of the plant [83], leaf thickness [134], leaf overlapping [69], and plant species and cultivars [72,132] are other properties that can influence relations between color parameters and plant characteristics. Measurement conditions (e.g., distance between camera and plant canopy [83] or measurements on the leaf level/on the whole plant level [44]) can also influence this efficiency. Finally, in some cases, this efficiency can dramatically decrease when using total datasets that include plants with different characteristics, e.g., determination coefficients for regressions describing relations between b* and leaf nitrogen concentration are 0.58–0.86 for datasets of rice plants with specific growth stages and 0.12 for datasets of rice plants with all growth stages (total dataset) [134].

Thus, the variability of relations between specific color parameters and specific plant characteristics can be a factor that restricts the efficiency of using these parameters in plant remote sensing. Revealing and providing optimal measurement conditions or searching and using color parameters that have stable relations with plant characteristics are potential ways to eliminate this restriction.

## 5. Transformation of Color Images to Multispectral and Hyperspectral Images

It is known [17,18] that the interaction with plant tissues can strongly change light spectra. Chlorophylls, carotenoids, and anthocyanins absorb light in the visible spectral region [20,22,26] (characteristic of internal leaf structure influence absorption and scattering of the NIR light [18,28]), and water content is related to SWIR light absorption [27]. As a result, specific plant characteristics are considered to be related to the light reflectance in specific narrow spectral bands. Thus, plant remote sensing based on multispectral and hyperspectral imaging is widely used to detect the action of abiotic stressors and phytopathogens and to estimate the growth rate, development, and productivity [12,17,25,31]. There are numerous methods devoted to analysis of results of both variants of imaging [12,28]. However, multispectral and hyperspectral cameras are technically complex and have a high cost [28,30], meaning that the estimation of parameters of multispectral and hyperspectral imaging based on the parameters of simple and low-cost RGB imaging can be a very important problem.

**Figure 2:**
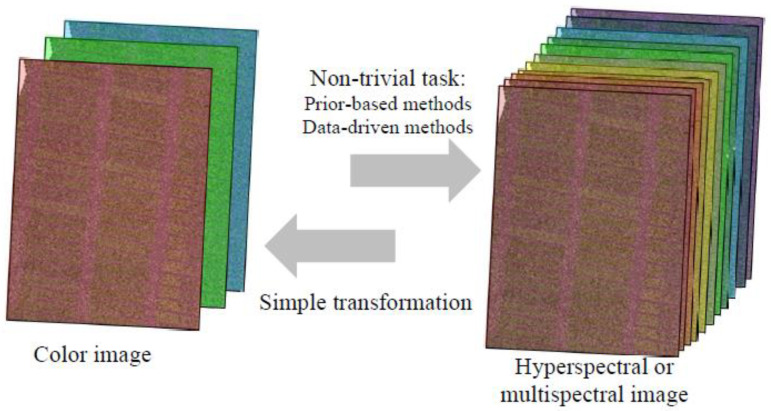
General schema of transformation between the color (RGB) image and hyperspectral or multispectral image. The color image is based on reflectance at three broad spectral bands (red, green, and blue); the hyperspectral or multispectral image is based on reflectance at the sequence of the narrow spectral bands or at several separate narrow spectral bands, respectively.

It is known that the transformation of a hyperspectral image in a color image is a simple task because it requires the reduction of information [57]. However, the inverse transformation from a color image to a hyperspectral or multispectral image is not a trivial task (Figure 2). The methods of reconstruction of hyperspectral or multispectral images from color images are dynamically developed and can be divided into the following two groups [56]:

(1) Prior-based methods (for example, dictionary learning [153,154], manifold learning [155] and the Gaussian process [156]) use statistical information such as spatial structure similarity, spectral correlation, sparsity, and others. These methods are mainly based on creating libraries of elementary reflectance spectra that form a total reflectance spectrum (or reflectance in specific narrow spectral bands) and weights of these spectra at different parameters of color images. The libraries provide coefficients for transformation of the RGB image to the plausible hyperspectral (or multispectral) image.

(2) Data-driven methods are based on deep machine learning using different neural networks, including BPNN (back propagation neural network) [157], HSCNN (hybrid Siamese convolutional neural network) [158], GAN (generative adversarial network) [159], and others. These methods do not require prior information (or hypothesis) about elementary spectra forming the total reflectance (or reflectance in specific narrow spectral bands); however, they need large datasets for training, validation, and testing.

In general, these methods can be used for reconstructions of hyperspectral images that include plants, landscapes, various anthropogenic artifacts, and other objects [56,153,154]; however, this “universal” reconstruction is difficult because the spectra of objects in the image can be strongly varied, disrupting the accuracy of the reconstructed total reflectance spectra. Alternatively, the reconstruction of hyperspectral or multispectral images of only plants or fruits can increase the accuracy of this reconstruction [153,154].

Particularly, Gong et al. [160] investigated the efficiency of the reconstruction of the hyperspectral image of plant leaves from color images based on using the BPNN and pseudo-inverse methods. It was shown that using the BPNN method provided a more accurate reconstruction than the pseudo-inverse method. The maximal error of the reconstruction was observed in the 530–560 nm spectral region. These hyperspectral images reconstructed from color images can be used as input into a regression model to estimate the chlorophyll content in each pixel of the image [160]; however, sensitivity of the reconstructed reflectance spectra to the chlorophyll content is lower than this sensitivity of measured reflectance spectra.

Using the MHPCG-Net (multimodal hyperspectral point cloud generation network) forms the reflectance spectra with a 10 nm spectral resolution, depth map, and 3D point cloud based on color images [161]; these parameters provide both spectral information and information about physical geometry of investigated plants. This method can be used for investigations of whole plants and their parts.

The reflectance spectra of fruits are also analyzed by the described methods. Particularly, hyperspectral images of tomato fruit can be reconstructed from color images based on using HSCNN [158]. The reconstructed spectra (using the random forest model) predict the lycopene content and ratio of soluble solid content to total titratable acidity.

Thus, there are methods of reconstruction of plant reflectance spectra based on their color images; however, these reconstructions are not unique ways to estimate parameters of multispectral and hyperspectral imaging based on RGB imaging. Particularly, the analysis of relations between color parameters and narrowband reflectance indices is another way of this estimation. Narrowband reflectance indices are known to be widely used for the estimation of photosynthesis activity, pigment content, de-epoxidation of carotenoids, LAI, biomass, water and nitrogen content, and others [17,25,28], meaning that the reconstruction of these indices based on color parameters can be an effective tool for plant remote sensing.

It is known that broadband indices can be related to plant characteristics [42,152,162] and, particularly, to narrowband reflectance indices [152]. The results potentially show that color parameters (including color indices) can be used for the estimation of narrowband reflectance indices.

The sparse dictionary method can be used for the reconstruction of reflectance in red and NIR narrow spectral bands from color images [163]. Further, this reconstruction is used to calculate NDVI. There are works that reveal relations of color indices or color coordinates to narrowband spectral indices (Table 3) being sensitive to biomass and LAI (NDVI, WDRVI, VARI, SRVI, and SRRE), chlorophyll content (CIgreen and CCCI), senescence (NDVI), or flower forming [44,52,113,131].

Thus, the transformation of color images to hyperspectral and multispectral images can strongly increase the efficiency and availability of plant remote sensing; however, further development of these methods remains a topical problem.

## 6. Conclusions and Perspectives

Methods of optical remote sensing are effective tools for the estimation of plant characteristics, with methods based on ecological monitoring, crop management, and plant protection. RGB imaging is a perspective optical method of plant remote sensing because RGB cameras are technically simple, low-cost, and, therefore, the most accessible. However, the effective use of plant RGB imaging requires the development of methods of analysis of color images to provide information about characteristics of plants.

There are different ways of color image analysis. Particularly, using color indices and color coordinates, which are related to the concentration of photosynthetic pigments, nitrogen and water contents, biomass, grain yield, LAI, senescence, action of water deficit, etc., is a perspective method for estimating plant characteristics. However, the high variability of efficiency of these parameters for estimating plant characteristics is an important problem of using RGB imaging because there are only some parameters that are stably effective. For example, VARI is stably related to the leaf nitrogen concentration or DGCI is stably related to the concentrations of chlorophyll a and carotenoids. In contrast, e.g., the determination coefficients for relations of the color coordinate R to the total chlorophyll content or leaf nitrogen concentration can be strongly ranging (R^2^ = 0.08–0.94 or R^2^ = 0.05–0.99, respectively). This variability of efficiency is probably the main limitation of using color parameters for estimating plant characteristics. There are methods to increase the efficiency of using color coordinates or indices for estimating plant characteristics that can be, particularly, based on using PCA, regression and canopy volume models, texture analysis, machine learning, and many other tools of the complex analysis of color parameters.

The development of methods of reconstruction of hyperspectral and multispectral images (including reconstruction of narrowband reflectance indices) based on color images is an alternative perspective way of increasing informativity of plant RGB imaging because reflectance spectra and narrowband reflectance indices can be strongly related to specific characteristics of plants. However, the efficiency of these methods can be strongly limited by the accuracy of the reconstruction of reflectance spectra, reflectance in specific spectral narrow bands, or narrowband reflectance indices.

Although all the noted ways of color image analysis are actively used, they have limitations and require further development to provide increasing analysis accuracy and extend investigated plant characteristics. Some potential perspectives of this development should be noted as follows. (i) Revealing new color parameters with maximum and stable efficiency of estimating specific plant characteristics; searching conditions to maximize this efficiency. (ii) Development of methods of the complex analysis of color and spatial (particularly, texture) parameters using regression models, crop surface models, machine learning, and other approaches. It is probable that the simultaneous use of additional methods of remote sensing of plant and, maybe, soils can improve the efficiency of this analysis (see, e.g., work [164], which is devoted to the development of complex methods of estimating soil organic carbon at plant cultivation). (iii) Development and analysis of radiation transfer models, which describe light absorption, transmission, and reflectance in plant leaves and canopy. Our review is not focused on the description of the models; however, radiation transfer models [165,166,167] are effectively used as a tool to analyze plant reflectance and to interpret the results of multispectral and hyperspectral imaging of terrestrial plants. It is probable that these models can also be used to analyze the results of plant RGB imaging.

## Figures and Tables

**Table 2:** Plant characteristics (related to color parameters) and abiotic stressors that influence these parameters.

Name of Color Parameter	Equation	Plant Characteristic/Influencing Factor	Reference
Red coordinate	R	***Total chlorophyll content*** ***(R^2^ = 0.08–0.94)***	[37,92,94,95,96,98]
		Chlorophyll a (R^2^ = 0.45) Chlorophyll b (R^2^ = 0.4) Carotenoids (R^2^ = 0.45)	[37]
		***SPAD (R^2^ = 0.08–0.92)***	[9,83,93,99]
		**Plant nitrogen concentration (R^2^ = 0.91)**	[100]
		***Leaf nitrogen concentration (R^2^ = 0.05–0.99)***	[91,96]
		Nitrogen fertilization (R^2^ = 0.69)	[9]
		***N-nutrient index (R^2^ = 0.08–0.95)***	[96]
		Nitrogen deficit	[13]
		***Fresh biomass (R^2^ = 0.29–0.76)*** Dry biomass (R^2^ = 0.30–0.67)	[101]
		***Aboveground biomass (R^2^ = 0.34–0.82)***	[5,6,96,102]
		***LAI (R^2^ = 0.26–0.72)***	[101,102]
		Plant height (R^2^ = 0.17–0.55)	[101]
		***Grain yield (R^2^ = 0.65–0.96)***	[3,38]
		Protein content (R^2^ = 0.65) Oil content (R^2^ = 0.46) Protein plus oil content (R^2^ = 0.53) 1000-seed weight (R^2^ = 0.41)	[53]
		Water deficit	[13]
Green coordinate	G	***Total chlorophyll (R^2^ = 0.22–0.94)***	[9,37,91,92,95,96,98]
		Chlorophyll a (R^2^ = 0.67) Chlorophyll b (R^2^ = 0.62) Carotenoids (R^2^ = 0.67)	[37]
		***SPAD (R^2^ = 0.37–0.90)***	[9,53,66,83,93,99]
		***Leaf nitrogen concentration (R^2^ = 0.36–0.92)***	[91,96]
		Plant nitrogen concentration (R^2^ = 0.45–0.54) Sap nitrate concentration (R^2^ = 0.46)	[66]
		Nitrogen fertilization (R^2^ = 0.69)	[9]
		***N-nutrient index (R^2^ = 0.46–0.98)***	[96]
		Nitrogen deficit	[13]
		***Canopy biomass (R^2^ = 0.49–0.72)***	[66]
		***Aboveground biomass (R^2^ = 0.59–0.96)***	[5,6,96]
		***Grain yield (R^2^ = 0.39–99)***	[3,38]
		Protein content (R^2^ = 0.64) Oil content (R^2^ = 0.45) Protein plus oil content (R^2^ = 0.53) 1000-seed weight (R^2^ = 0.39)	[53]
		Water deficit	[13]
Blue coordinate	B	Carotenoids (R^2^ = 0.36)	[37]
		SPAD (R^2^ = 0.04–0.64)	[83,93]
		***Plant nitrogen concentration (R^2^ = 0.89)***	[100]
		Protein content (R^2^ = 0.62) Oil content (R^2^ = 0.45) Protein plus oil content (R^2^ = 0.50)	[53]
		***Grain yield (R^2^ = 0.47–0.97)***	[3]
		Aboveground biomass (R^2^ = 0.49)	[6]
		Plant water content (R^2^ = 0.48)	[102]
		Water deficit	[13]
Normalized red coordinate	$r=\frac{R}{R+G+B}$	***Total chlorophyll content (R^2^ = 0.03–0.91)***	[94,95,96]
		**Chlorophyll a (R^2^ = 0.74)** Chlorophyll b (R^2^ = 0.67) **Total chlorophyll (R^2^ = 0.74)** **Carotenoids (R^2^ = 0.76)**	[37]
		***SPAD (R^2^ = 0.03–0.8)***	[68,83,92,93,97,99,103]
		**Sap nitrate concentration (R^2^ = 0.78)**	[103]
		Plant nitrogen concentration (R^2^ = 0.48–0.67)	[104,105]
		Shoot nitrogen concentration (R^2^ = 0.63)	[103]
		***Leaf nitrogen concentration (R^2^ = 0.04–0.99)***	[96,105]
		***N-nutrient index (R^2^ = 0.03–0.96)***	[96]
		Nitrogen uptake (R^2^ = 0.34–0.41)	[96]
		***Aboveground biomass (R^2^ = 0.05–0.84)***	[6,96,102]
		LAI (R^2^ = 0.42–0.55)	[102]
		Plant height (R^2^ = 0.25–0.52)	[102]
		***Grain yield (R^2^ = 0.01–0.94)***	[3,4]
		Plant water content (R^2^ = 0.46–0.62)	[102]
		Water deficit	[13]
Normalized green coordinate	$g=\frac{G}{R+G+B}$	***Chlorophyll content (R^2^ = 0.21–0.81)***	[96]
		SPAD (R^2^ = 0.01–0.62)	[83,93,99,103,106]
		Plant nitrogen concentration (R^2^ = 0.29–0.61)	[69,104,105,106,107]
		***Leaf nitrogen concentration (R^2^ = 0.24–0.99)***	[96,105]
		Nitrate concentration of leafstalk (R^2^ = 0.62)	[106]
		Stem nitrate concentration (R^2^ = 0.62)	[107]
		Sap nitrate concentration (R^2^ = 0.68)	[103]
		***N-nutrient index (R^2^ = 0.27–0.94)***	[96]
		Shoot nitrogen accumulation (R^2^ = 0.56)	[108]
		Shoot nitrogen concentration (R^2^ = 0.68)	[103]
		**Inorganic nitrogen in soil (R^2^ = 0.76)**	[106]
		***Aboveground biomass (R^2^ = 0.02–0.79)***	[5,69,96]
		***Dry mass (R^2^ = 0.3–0.86)***	[108,109,110]
		LAI (R^2^ = 0.59)	[69,108]
		Plant height (R^2^ = 0.24–0.44)	[102]
		***Grain yield (R^2^ = 0.3–0.89)***	[3,107,109]
Normalized blue coordinate	$b=\frac{B}{R+G+B}$	***Total chlorophyll content (R^2^ = 0.01–0.90)***	[95,96,102]
		***SPAD (R^2^ = 0.03–0.71)***	[68,83,93,97,103]
		***Plant nitrogen concentration (R^2^ = 0.03–0.77)***	[102,104,105]
		Leaf nitrogen concentration (R^2^ 0.11–0.69)	[96,105]
		Shoot nitrogen concentration (R^2^ = 0.55)	[103]
		Sap nitrate concentration (R^2^ = 0.53)	[103]
		Nitrogen deficit	[13]
		N-nutrient index (R^2^ = 0.18–0.60)	[96]
		Aboveground biomass (R^2^ = 0.36–0.57)	[5,6]
		LAI (R^2^ = 0.03–0.48)	[102]
		***Grain yield (R^2^ = 0.06–0.86)***	[3,4]
		Water deficit	[13]
Red–green sum index (RGSI)	RGSI=R+G	***Total chlorophyll content (R^2^ = 0.72–0.87)***	[92,94]
		SPAD (R^2^ = 0.57–0.59)	[92,99]
Red–blue sum index (RBSI)	RBSI=R+B	Total chlorophyll content (R^2^ = 0.53)	[92]
		SPAD (R^2^ = 0.43–0.54)	[92,99]
Normalized red–blue sum index (NRBSI)	NRBSI=r+b	**Plant nitrogen concentration (R^2^ = 0.7)** **Leaf nitrogen concentration (R^2^ = 0.7)**	[105]
Green–blue sum index (GBSI)	GBSI=G+B	SPAD (R^2^ = 0.42)	[99]
Red-green blue sum index (RGBSI)	RGBSI=R+G+B	**Total chlorophyll content (R^2^ = 0.71)**	[92]
		SPAD (R^2^ = 0.54–0.64)	[92,99]
Difference BG-R index (DBGRI)	DBGRI=B+G−R	SPAD (R^2^ = 0.39)	[92]
Normalized Difference BG-R index (NDBGRI)	$NDBGRI=\frac{B+G-R}{B+G+R}$	**Chlorophyll a (R^2^ = 0.81–0.93)** **Chlorophyll b (R^2^ = 0.8–0.93)** **Carotenoids (R^2^ = 0.78–0.93)** ***Nitrogen content (R^2^ = 0.69–0.78)***	[44]
Red minus green	RMG=R−G	SPAD (R^2^ = 0.56)	[99]
Normalized green minus red index (NGMR)	NGMR=g−r	Chlorophyll a (R^2^ = 0.36–0.62) Chlorophyll b (R^2^ = 0.33–0.6) Carotenoids (R^2^ = 0.28–0.56)	[44]
Green minus red index (GMR)	GMR=G−R	SPAD (R^2^ = 0.36)	[9]
		***Plant nitrogen concentration (R^2^ = 0.46–0.93)***	[69,104]
		Nitrogen fertilization (R^2^ = 0.48)	[9]
		**LAI (R^2^ = 0.83–0.95)**	[69]
		**Aboveground biomass (R^2^ = 0.79–0.93)**	[69]
Normalized green minus blue (NGMB)	NGMB=g−b	Plant nitrogen concentration (R^2^ = 0.6) Leaf nitrogen concentration (R^2^ = 0.6)	[105]
		***Total chlorophyll content (R^2^ = 0.26–0.79)***	[95]
Green minus blue (GMB)	GMB=G−B	***Total chlorophyll content (R^2^ = 0.52–0.96)***	[9,95]
		***SPAD (R^2^ = 0.37–0.83)***	[9,83]
		**Nitrogen fertilization (R^2^ = 0.77)**	[9]
Normalized red minus blue index (NRMB)	NRMB=r−b	***Total chlorophyll content (R^2^ = 0.44–0.9)***	[95]
Red minus blue index (RMB)	RMB=R−B	***Total chlorophyll content (R^2^ = 0.44–0.99)***	[9,92,95,111]
		Chlorophyll a (R^2^ = 0.67) Chlorophyll b (R^2^ = 0.55)	[111]
		SPAD (R^2^ = 0.32–0.62)	[9,83,92,99,111]
		Leaf nitrogen content (R^2^ = 0.6)	[111]
		**Nitrogen fertilization (R^2^ = 0.76)**	[9]
Green–blue simple ratio (GB)	$GB=\frac{G}{B}$	***SPAD (R^2^ = 0.18–0.82)***	[112]
		**Plant nitrogen concentration (R^2^ = 0.7)** **Leaf nitrogen concentration (R^2^ = 0.7)**	[105]
		Aboveground biomass (R^2^ = 0.57)	[5]
Blue–green simple ratio (BG)	$BG=\frac{B}{G}$	***SPAD (R^2^ = 0.00–0.74)***	[83]
		**Fresh biomass (R^2^ = 0.92–0.94)** **Dry biomass (R^2^ = 0.74–0.85)** **LAI (R^2^ = 0.79–0.94)** **Plant height (R^2^ = 0.74–0.90)**	[101]
Red–blue simple ratio (RB)	$RB=\frac{R}{B}$	***Plant nitrogen concentration (R^2^ = 0.00–0.77)***	[104,105]
		Leaf nitrogen concentration (R^2^ = 0.6)	[105]
		**Fresh biomass (R^2^ = 0.77–0.94)** ***Dry biomass (R^2^ = 0.64–0.90)***	[101]
		Aboveground biomass (R^2^ = 0.47)	[5]
		**LAI (R^2^ = 0.76–0.88)** ***Plant height (R^2^ = 0.61–0.86)***	[101]
Blue–red simple ratio (BR)	$BR=\frac{B}{R}$	***Total chlorophyll content (R^2^ = 0.09–0.97)***	[96,111]
		Chlorophyll a (R^2^ = 0.6) Chlorophyll b (R^2^ = 0.45) SPAD (R^2^ = 0.48)	[111]
		***Leaf nitrogen content (R^2^ = 0.05–0.96)***	[36,96,111]
		Total canopy nitrogen content (R^2^ = 0.55)	[36]
		***N-nutrient level (R^2^ = 0.12–0.92)***	[96]
		***Aboveground biomass (R^2^ = 0.48–0.84)***	[96]
Green–red simple ratio (GR)	$GR=\frac{G}{R}$	***Total chlorophyll content (R^2^ = 0.01–0.92)***	[94,96]
		***Chlorophyll a (R^2^ = 0.69–0.89)*** ***Chlorophyll b (R^2^ = 0.68–0.89)*** ***Carotenoids (R^2^ = 0.64–0.88)***	[44]
		***SPAD (R^2^ = 0.04–0.91)***	[68,83,112,113]
		***Aboveground biomass (R^2^ = 0.01–0.85)***	[50,69,96]
		***LAI (R^2^ = 0.66–0.93)***	[69]
		***Plant nitrogen concentration (R^2^ = 0.55–0.92)***	[69,104,105]
		***Leaf nitrogen concentration (R^2^ = 0.12–0.99)***	[96,105]
		***N-nutrient index (R^2^ = 0.11–0.95)***	[96]
Red–green ratio index (RGRI)	$RGRI=\frac{R}{G}$	Canopy temperature	[54]
		**Leaf nitrogen concentration (R^2^ = 0.7–0.87)**	[47,105]
		***Plant nitrogen concentration (R^2^ = 0.1–0.7)***	[101,105]
		**Flower number (R^2^ = 0.83)**	[52]
Normalized difference red–blue index (NDRBI), (alternative name is Kawashima index, IKAW)	$NDRBI=\frac{R-B}{R+B}$	***Total chlorophyll content (R^2^ = 0.33–0.9)***	[94,95,111]
		Chlorophyll a (R^2^ = 0.59) Chlorophyll b (R^2^ = 0.44)	[111]
		***SPAD (R^2^ = 0.2–0.84)***	[111,112,114,115]
		***Leaf nitrogen concentration (R^2^ = 0.51–0.92)***	[47,105,111]
		Plant nitrogen concentration (R^2^ = 0.6)	[105]
		Aboveground biomass (R^2^ = 0.48–0.50)	[5,116]
		**LAI (R^2^ = 0.71)**	[117]
		Grain yield (R^2^ = 0.25–0.59)	[114]
Normalized green–blue difference index; normalized difference green–blue index (NGBDI, NDGBI)	$NGBDI=\frac{G-B}{G+B}$	***Total chlorophyll content (R^2^ = 0.29–0.85)***	[95]
		***SPAD (R^2^ = 0.00–0.71)***	[83]
Normalized green–red difference index (NGRDI)	$NGRDI=\frac{G-R}{G+R}$	Total leaf chlorophyll (R^2^ = 0.62)	[118]
		**Chlorophyll a (R^2^ = 0.72–0.89)** **Chlorophyll b (R^2^ = 0.71–0.89)** ***Carotenoids (R^2^ = 0.68–0.89)***	[44]
		***SPAD (R^2^ = 0.05–0.84)***	[83,112,118,119]
		***Plant nitrogen concentration (R^2^ = 0.46–0.79)***	[104,105,120]
		***Leaf nitrogen concentration (R^2^ = 0.51–0.87)***	[47,105,120]
		Leaf nitrogen accumulation (R^2^ = 0.5)	[43]
		Aboveground biomass (R^2^ = 0.39–0.56)	[40,50,116,121]
		Aboveground dry biomass (R^2^ = 0.3–0.55)	[41]
		***Dry biomass (R^2^ = 0.0–0.92)***	[72,122,123]
		Leaf dry matter (R^2^ = 0.53)	[43]
		***LAI (R^2^ = 0.18–0.74)***	[43,102,117,124]
		Surface of an individual plant	[125]
		***Vegetation cover (R^2^ = 0.05–0.81)***	[45]
		Stem diameter	[125]
		Plant height (R^2^ = 0.27–0.53)	[102,125]
		Plant water content (R^2^ = 0.37–0.50)	[102]
		Grain yield (R^2^ = 0.26–0.59)	[124]
		**Flower number (R^2^ = 0.83)**	[52]
Woebbecke’s indices (WI)	$WI=\frac{G-B}{\left|R-G\right|}$	***SPAD (R^2^ = 0.17–0.85)***	[112]
Ku’s index (KI)	$KI=\frac{R}{R-B}$	***Total chlorophyll (R^2^ = 0.33–0.99)***	[9]
Simple ratio intensity R-GB (SRrgb)	$SRrgb=\frac{R}{G+B}$	**Chlorophyll a (R^2^ = 0.82–0.93)** **Chlorophyll b (R^2^ = 0.81–0.93)** **Carotenoids (R^2^ = 0.79–0.93)** ***Leaf nitrogen concentration (R^2^ = 0.69–0.79)***	[44]
Normalized difference index (NDI)	$NDI=\frac{r-g}{r+g+0.01}$	**Chlorophyll a (R^2^ = 0.72–0.9)** **Chlorophyll b (R^2^ = 0.71–0.9)** ***Carotenoids (R^2^ = 0.67–0.89)***	[44]
		Dry mass (R^2^ = 0.48) LAI (R^2^ = 0.52) Shoot nitrogen accumulation (R^2^ = 0.56)	[108]
Soil adjusted vegetation index green (RGB) (SAVIgreen)	${SAVI}_{green}=\frac{\left(1+L)(g-r\right)}{\left(g+r\right)+L}$, L = 0.5	**Chlorophyll a (R^2^ = 0.7–0.89)** ***Chlorophyll b (R^2^ = 0.69–0.88)*** ***Carotenoids (R^2^ = 0.65–0.88)***	[44]
Optimized soil adjusted vegetation index green (RGB) (OSAVIgreen)	${OSAVI}_{green}=\frac{1.5\cdot (g-r)}{g+r+0.16}$	**Chlorophyll a (R^2^ = 0.71–0.89)** **Chlorophyll b (R^2^ = 0.7–0.89)** ***Carotenoids (R^2^ = 0.67–0.88)***	[44]
Enhanced vegetation index green (RGB) (EVIgreen)	${EVI}_{green}=\frac{2.5\cdot (g-r)}{g+6\cdot r-7.5\cdot b+1}$	**Chlorophyll a (R^2^ = 0.81–0.92)** **Chlorophyll b (R^2^ = 0.8–0.92)** **Carotenoids (R^2^ = 0.79–0.92)** ***Leaf nitrogen concentration (R^2^ = 0.69–0.77)***	[44]
Enhanced vegetation index 2 green (RGB) (EVI2green)	${EVI2}_{green}=\frac{2.5\cdot (g-r)}{g+2.4\cdot r+1}$	***Chlorophyll a (R^2^ = 0.69–0.89)*** ***Chlorophyll b (R^2^ = 0.68–0.88)*** ***Carotenoids (R^2^ = 0.66–0.88)***	[44]
Principal component analysis Pagola’s index (I_PCA_)	$I_{pca}=0.7582\left|r-b\right|-$ $-0.1168\left|r-g\right|+0.6414\left|g-b\right|$	***SPAD (R^2^ = 0.1–0.88)*** Grain yield (R^2^ = 0.35–0.59)	[114]
Principal component analysis Saberioon’s index (I_PCAS_)	$I_{PCAS}=0.994\left|R-B\right|+$ $+0.914\left|G-R\right|+0.961\left|G-B\right|$	SPAD (R^2^ = 0.62)	[126]
		**Aboveground biomass (R^2^ = 0.78)**	[5]
Red–green fit index (RGFI)	RGFI=2·G−0.924·R− −44.851−B	**SPAD (R^2^ = 0.94)**	[119]
Blue–green fit index (BGFI)	BGFI=2·G−R− −73.645−0.71·B	SPAD (R^2^ = 0.62)	[119]
Normalized excess green index (NExG)	NExG=2·g−r−b	***Canopy-to-air temperature difference (R^2^ = 0.47–0.8)*** ***Crop water stress index (R^2^ = 0.63–0.8)*** ***Canopy temperature (R^2^ = 0.66–0.73)*** **Leaf water potential (R^2^ = 0.85–0.87)** Sap flow (R^2^ = 0.62)	[55]
		SPAD (R^2^ = 0.45)	[126]
		Plant nitrogen concentration (R^2^ = 0.28–0.68)	[105,120]
		Leaf nitrogen concentration (R^2^ = 0.26–0.65)	[105,120]
		Aboveground biomass (R^2^ = 0.47)	[5]
		***Fresh biomass (R^2^ = 0.48–0.88)*** ***Dry biomass (R^2^ = 0.27–0.81)***	[101]
		Global primary productivity (GPP)	[46]
		***LAI (R^2^ = 0.09–0.88)***	[101,124]
		Surface of an individual plant	[125]
		***Vegetation cover (R^2^ = 0.03–0.77)***	[45]
		Plant height (R^2^ = 0.53–0.69)	[101,125]
		Stem diameter	[125]
Excess green index (ExG)	ExG=2·G−R−B	***SPAD (R^2^ = 0.34–0.86)***	[83,119]
		Flower number (R^2^ = 0.58)	[52]
Excess red index (ExR)	ExR=1.4·g−r	***Plant nitrogen concentration (R^2^ = 0.52–0.72)***	[104,105]
		**Leaf nitrogen concentration (R^2^ = 0.7)**	[105]
		Leaf nitrogen accumulation (R^2^ = 0.49)	[43]
		Aboveground biomass (R^2^ = 0.40–0.56)	[102,116]
		***LAI (R^2^ = 0.16–0.8)***	[43,102,117,124]
		Leaf dry matter (R^2^ = 0.52)	[43]
		Surface of an individual plant Stem diameter	[125]
		Plant height (R^2^ = 0.26–0.52)	[102,125]
		Plant water content (R^2^ = 0.40–0.52)	[102]
		Grain yield (R^2^ = 0.26–0.58)	[124]
Excess blue vegetation index (ExB)	ExB=1.4·b−g	Aboveground biomass (R^2^ = 0.57)	[5]
		**Plant nitrogen concentration (R^2^ = 0.7)** **Leaf nitrogen concentration (R^2^ = 0.7)**	[105]
Normalized excess green minus excess red (ExGR)	NExGR=NExG−NExR	SPAD (R^2^ = 0.44)	[126]
		Plant nitrogen concentration (R^2^ = 0.6) Leaf nitrogen concentration (R^2^ = 0.6)	[105]
		LAI (R^2^ = 0.09–0.65)	[124]
		Aboveground biomass (R^2^ = 0.39)	[5]
		Surface of an individual plant Plant height Stem diameter	[125]
Excess green minus excess red (ExGR)	ExGR=ExG−ExR	***SPAD (R^2^ = 0.09–0.72)***	[112]
Green leaf index (GLI)	$GLI=\frac{2\cdot G-R-B}{2\cdot G+R+B}$	***SPAD (R^2^ = 0.00–0.79)***	[83,118,119,127]
		Total leaf chlorophyll (R^2^ = 0.64)	[118]
		Leaf nitrogen concentration (R^2^ = 0.6) Plant nitrogen concentration (R^2^ = 0.6)	[105]
		***Aboveground biomass (R^2^ = 0.49–0.74)***	[5,50]
		Dry biomass (R^2^ = 0.33–0.36)	[123]
		LAI (R^2^ = 0.07–0.58)	[124]
		Surface of an individual plant Stem diameter	[125]
		Plant height (R^2^ = 0.24–0.44)	[102,125]
		Flower number (R^2^ = 0.37)	[52]
		Water damage in field	[128]
Modified green–red vegetation index (MGRVI)	$MGRVI=\frac{G^{2}-R^{2}}{G^{2}+R^{2}}$	Plant nitrogen concentration (R^2^ = 0.6) Leaf nitrogen concentration (R^2^ = 0.6)	[105]
		Aboveground biomass (R^2^ = 0.40–0.56)	[50,102,116]
		Dry biomass (R^2^ = 0.53–0.59)	[122]
		***LAI (R^2^ = 0.45–0.80)***	[102,117]
		Plant height (R^2^ = 0.27–0.53)	[102]
		Plant water content (R^2^ = 0.37–0.48)	[102]
		**Flower number (R^2^ = 0.83)**	[52]
Red–green blue vegetation index (RGBVI)	$RGBVI=\frac{G^{2}-R\cdot B}{G^{2}+R\cdot B}$	SPAD (R^2^ = 0.18–0.53)	[112]
		Plant nitrogen concentration (R^2^ = 0.6) Leaf nitrogen concentration (R^2^ = 0.6)	[105]
		Fresh biomass (R^2^ = 0.21–0.55)	[129]
		Dry biomass (R^2^ = 0.44)	[122]
Color index of vegetation (CIVE)	CIVE=0.441·R−0.881·G+ +0.385·B+18.78745	**Aboveground biomass (R^2^ = 0.72)**	[5]
		Flower number (R^2^ = 0.59)	[52]
Color index of vegetation (CIVE)	CIVE=0.441·r−0.881·g+ +0.385·b+18.78745	Plant height (R^2^ = 0.24–0.46)	[102]
Vegetative index (VEG)	$VEG=\frac{G}{R^{a}B^{1-a}}a=0.667$	***SPAD (R^2^ = 0.17–0.84)***	[112]
		***LAI (R^2^ = 0.5–0.8)***	[117]
		***Aboveground biomass (R^2^ = 0.4–0.71)***	[5,50]
		Flower number (R^2^ = 0.44)	[52]
True color vegetation index (TCVI)	$TCVI=\;=\frac{1.4\cdot 2\cdot \left(R-B\right)}{2\cdot R-G-2\cdot B+255\cdot 0.4}$	**Leaf nitrogen concentration (R^2^ = 0.81–0.91)**	[47]
Visible atmospherically resistance index (VARI)	$VARI=\frac{G-R}{G+R-B}$	Total leaf chlorophyll (R^2^ = 0.61)	[118]
		**Chlorophyll a (R^2^ = 0.78–0.92)** **Chlorophyll b (R^2^ = 0.77–0.92)** **Carotenoids (R^2^ = 0.75–0.92)**	[44]
		***Plant nitrogen concentration (R^2^ = 0.59–0.77)***	[104,105]
		**Leaf nitrogen concentration (R^2^ = 0.7–0.89)**	[44,47,105]
		Leaf nitrogen accumulation (R^2^ = 0.61)	[43]
		***SPAD (R^2^ = 0.18–0.75)***	[112,118]
		Aboveground biomass (R^2^ = 0.16–0.62)	[6,50,102,116]
		Leaf dry matter (R^2^ = 0.64)	[43]
		Dry biomass (R^2^ =0.57–0.63)	[123]
		***LAI (R^2^ = 0.23–0.77)***	[43,102,117,124]
		Surface of an individual plant Stem diameter	[125]
		Plant height (R^2^ = 0.27–0.52)	[102,125]
		Plant water content (R^2^ = 0.38–0.52)	[102]
		***Grain yield (R^2^ = 0.28–0.71)***	[124]
		**Flower number (R^2^ = 0.81)**	[52]
Visible atmospherically resistance index (VARI) by Sakamoto	$VARIs=\frac{G-R}{G+R}$	***Plant length (R^2^ = 0.23–0.98)*** ***Total dry weight (R^2^ = 0.06–0.97)***	[130]
		***LAI (R^2^ = 0.62–0.98)***	[130,131]
Combination (COM)	COM=0.25·NExG+0.3··NExGR+ +0.33·CIVE+0.12·VEG	**Aboveground biomass (R^2^ = 0.72)**	[5]
Ch_OL_	$\log sig(\frac{G-\frac{R}{3}-\frac{B}{3}}{255})$	***Total chlorophyll content (R^2^ = 0.48–0.94)***	[94,98]
Coordinates of the HSI model	Hue	**Total chlorophyll content (R^2^ = 0.71)**	[91]
		**Leaf nitrogen concentration (R^2^ = 0.77)**	[91]
		Nitrogen deficit Water deficit	[13]
	Saturation	Water deficit	[13]
	Intensity	***SPAD (R^2^ = 0.08–0.92)***	[83]
		***Plant nitrogen concentration (R^2^ = 0.49–0.76)***	[104]
		Nitrogen deficit Water deficit	[13]
		Aboveground biomass (R^2^ = 0.57)	[5]
Coordinates of the HSB model	Hue	Total chlorophyll (R^2^ = 0.48)	[37]
		***Chlorophyll a (R^2^ = 0.49–0.92)*** ***Chlorophyll b (R^2^ = 0.4–0.92)*** ***Carotenoids (R^2^ = 0.49–0.92)***	[37,44]
		***SPAD (R^2^ = 0.65–0.76)***	[68]
		**Leaf nitrogen concentration (R^2^ = 0.71–0.79)**	[44]
		Protein content (R^2^ = 0.64) Oil content (R^2^ = 0.50) Protein plus oil content (R^2^ = 0.49) 1000-seed weight (R^2^ = 0.43)	[53]
	Saturation	**Total chlorophyll (R^2^ = 0.77)** **Chlorophyll a (R^2^ = 0.77)** **Chlorophyll b (R^2^ = 0.71)** **Carotenoids (R^2^ = 0.77)**	[37]
		Protein content (R^2^ = 0.69) Oil content (R^2^ = 0.47) Protein plus oil content (R^2^ = 0.58) 1000-seed weight (R^2^ = 0.40)	[53]
	Brightness	Total chlorophyll (R^2^ = 0.66) Chlorophyll a (R^2^ = 0.67) Chlorophyll b (R^2^ = 0.61) Carotenoids (R^2^ = 0.66)	[37]
		Protein content (R^2^ = 0.64) Oil content (R^2^ = 0.46) Protein plus oil content (R^2^ = 0.53) 1000-seed weight (R^2^ = 0.39)	[53]
Dark green color index (DGCI) on basis HSB model	$DGCI=\frac{\frac{Hue-60}{60}}{3}+$ $+\frac{\left(1-Saturation\right)}{3}+$ $+\frac{1-Brightness}{3}$	**Total chlorophyll (R^2^ = 0.71)**	[37]
		**Chlorophyll a (R^2^ = 0.71–0.86)** ***Chlorophyll b (R^2^ = 0.61–0.86)*** **Carotenoids (R^2^ = 0.71–0.86)**	[37,44]
		***SPAD (R^2^ = 0.16–0.94)***	[132]
		***Leaf nitrogen concentration (R^2^ = 0.18–0.86)***	[132]
		Nitrogen fertilization	[133]
		***Grain yield (R^2^ = 0.04–0.88)***	[132]
Coordinates of the L*a*b* model	$L^{*}$	Total chlorophyll content (R^2^ = 0.66–0.68)	[37,92]
		Chlorophyll a (R^2^ = 0.66) Chlorophyll b (R^2^ = 0.61) Carotenoids (R^2^ = 0.66)	[37]
		SPAD (R^2^ = 0.58–0.85)	[83,92]
	$a^{*}$	Total chlorophyll content (R^2^ = 0.38–0.39)	[37,92]
		Chlorophyll a (R^2^ = 0.38) Chlorophyll b (R^2^ = 0.38) Carotenoids (R^2^ = 0.37)	[37]
		SPAD (R^2^ = 0.44)	[92]
		N, P, Mg, and Fe deficit	[7]
	$b^{*}$	***Total chlorophyll content (R^2^ = 0.62–0.81)***	[37,92]
		**Chlorophyll a (R^2^ = 0.81)** **Chlorophyll b (R^2^ = 0.74)** **Carotenoids (R^2^ = 0.81)**	[37]
		***SPAD (R^2^ = 0.31–0.81)***	[83,92,134]
		***Leaf nitrogen concentration (R^2^ = 0.58–0.86)***	[134]
		Nitrogen fertilization	[135]
		Plant nitrogen concentration (R^2^ = 0.66–0.67)	[7,136]
Ratio of b* to a*	$\frac{b^{*}}{a^{*}}$	SPAD (R^2^ = 0.02–0.67)	[83,134]

R^2^ is the determination coefficient for the regression describing the relation between the color parameter and plant characteristic. These coefficients are directly provided in cited works or are calculated as squares of Pearson correlation coefficients (R^2^ for the linear regression). If several R^2^s are shown in the cited works (e.g., under different measuring conditions), the ranging R^2^ is included in this table. The determination coefficient is absent from this table if the relations between color parameters and plant characteristics are not investigated or if the Spearmen correlation coefficient is analyzed in cited works. Relations with minimum R^2^ ≥ 0.7 (for separate determination coefficients or their ranges) are marked by bold. Relations with maximum R^2^ ≥ 0.7 (for ranges of determination coefficients) are marked by bold and italics. Relations with maximum R^2^ < 0.36 are not included in this table because R^2^ = 0.36 approximately corresponds with the correlation coefficient with absolute value equaling to 0.6, i.e., R^2^ < 0.36 corresponds with the weak correlation. Non-marked relations are moderate and can be potentially used to further develop effective tools for estimation of plant characteristics (e.g., through the combination of several color parameters that have these relations).

**Table 3:** Color indices related to narrowband reflectance indices.

Color Indices	Narrowband Reflectance Indices	Reference
GR	Normalized Difference Vegetation Index (NDVI)	[44,113]
	Narrowband spectral indices Simple Ratio Vegetation Index (SRVI) Simple Ration Red Edge (SRRE) Normalized Difference Red Edge (NDRE) Canopy Chlorophyll Content Index (CCCI)	[44]
NGRDI	Spectral indices (R_944_ − R_758_)/(R_944_ + R_758_), R_944_/R_758_	[52]
	Narrowband spectral indices Simple Ratio Vegetation Index (SRVI) Simple Ration Red Edge (SRRE) Normalized Difference Red Edge (NDRE) Normalized Difference Vegetation Index (NDVI) Canopy Chlorophyll Content Index (CCCI)	[44]
ExG	Normalized Difference Vegetation Index (NDVI) Green Red Normalized Difference index (VARI) Simple ratio of NIR and Red (SR) Green Chlorophyll index (CI_green_)	[131]
	Spectral indices (R_944_ − R_758_)/(R_944_ + R_758_), R_944_/R_758_	[52]
VARI	Spectral indices (R_944_ − R_758_)/(R_944_ + R_758_), R_944_/R_758_	[52]
	Narrowband spectral indices Simple Ratio Vegetation Index (SRVI) Simple Ration Red Edge (SRRE) Normalized Difference Red Edge (NDRE) Normalized Difference Vegetation Index (NDVI) Canopy Chlorophyll Content Index (CCCI)	[44]
VARIs	Normalized Difference Vegetation Index (NDVI) Green Red Normalized Difference index (VARI) Simple ratio of NIR and Red (SR) Green Chlorophyll index (CI_green_)	[131]
NDBGRI	Narrowband spectral indices Simple Ratio Vegetation Index (SRVI) Simple Ration Red Edge (SRRE) Normalized Difference Red Edge (NDRE) Normalized Difference Vegetation Index (NDVI) Canopy Chlorophyll Content Index (CCCI)	[44]
GMR		
NDI		
SAVI_green_		
OSAVIgreen		
EVI_green_		
EVI2_green_		
Hue (HSB)		
DGCI (HSB)		
RGRI	Spectral indices (R_944_ − R_758_)/(R_944_ + R_758_), R_944_/R_758_	[52]
GLI		
VEG		
CIVE		
MGRVI		

The determination coefficients (R^2^) for the regressions describing relation between color indices and narrowband reflectance indices are about 0.50 and more (mostly 0.70–0.96) excluding GMR. The last color index is weakly related to narrowband reflectance indices in some cases (e.g., R^2^ could be 0.38 for GMR and SRRE [44]).
